# Effects of Risk Factors on Belizean Adolescents’ Academic Behaviors and Grit after Prolonged Absence During the COVID-19 Pandemic

**DOI:** 10.5334/cie.41

**Published:** 2022-06-17

**Authors:** Mathias Vairez, Frank Gomez, Carolyn Gentle-Genitty, Janeen Quiroz, Olga Manzanero

**Affiliations:** 1 University of Belize, BZ; 2 Maria Regina School, US; 3 Indiana University, US

**Keywords:** School Absenteeism, Risk Factors, Academic Behaviors, Adolescents, Belize, COVID-19, Grit

## Abstract

This causal-comparative study explored the effects of risk factors—family status, parental marital status, family income, and parent education level—on Belizean adolescents’ academic behaviors and grit (passion and perseverance in goal achievement) following prolonged absence during the COVID-19 pandemic. Data were collected online using a demographic survey, the Grit-S Scale (Duckworth & Quinn, 2009), coupled with eight additional items to measure academic behaviors (attendance, preparedness, attention, note-taking, participation, organization, use of out-of-school time, and homework completion and submission) for success (Farrington et al., 2012) from secondary and tertiary students in Belize. With rare exception, Belizean education took place in person before the pandemic. This changed to remote teaching and learning during the pandemic. Findings showed that adolescents from the defined risk factor of single-parent households experienced greater declines across all eight academic behaviors. Additionally, this effect was more pronounced for adolescents who experienced the loss of a parent from divorce or death of a parent. For grit, there were two key outcomes: (a) adolescents from nuclear and higher income families had slightly higher levels of grit; and (b) adolescents from parents with lower educational attainment had significantly higher levels of grit than their peers. Based on these findings, recommendations include more study of schools that invest in becoming trauma responsive when evaluating engagement and performance during prolonged absences. Future research should assess adolescents’ level of academic behaviors, grit, and other noncognitive factors.

## Introduction

A small country in Central America where the water is still fresh and the land is still pure is often the picture painted of Belize. Situated at the north end of Central America, Belize is located with Guatemala to the west and south, the Caribbean Sea to the east, and Mexico to the north. According to Rich et al. (2022), despite unusual fluctuation, under the 2020–2021 COVID-19 pandemic, tourism was the major foreign-exchange earner.

Belize is a densely populated nation with a stable democratic political system of governance. As of 2021, the estimated population was approximately 405,633, predominantly young, with almost 52% being 0–24 years old, and concentrated in Belize City (Belize World Factbook, 2021). Per the Human Development Index (2020), the life expectancy at birth in Belize is 74.6 years (an increase from the 2006 Human Development Index figures), with 13.1 expected years of schooling. The country is diverse within ethnic and racial groups: Mestizo (52.9%), Creole (25.9%), three groups of indigenous Maya, namely Yucatec, Mopan, and Q’eqchi’ (11.3%), an Afro-Indigenous Garifuna population (6.1%), East Indian (3.9%), Mennonite (3.6%), Caucasian (1.2%), Asian (1%), and other/unknown (1.5%). Likewise, Belize has great religious diversity: Roman Catholic (40.1%); Protestant (31.5%, which includes Pentecostal, Seventh Day Adventists, Anglican, Mennonite, Baptist, Methodist, Nazarene), Jehovah’s Witness (1.7%); other (10.5%, including Buddhist, Hindu, Mormon, Muslim, and Rastafarian), and none (15.5%) (Belize World Factbook, 2021). The country boasted an unemployment rate of 9% in 2017; yet according to a 2013 estimate, 41% of the population was living below the poverty line (Belize World Factbook, 2021).

According to the Belize Ministry of Education, three types of educational institutions deliver education services to Belizeans: government (owned and funded by government), government-aided (owned by religious or other groups but receiving subsidized government funding for salaries or services), and private (owned and funded by individual persons, denominations, or private groups). At government and government-aided institutions tuition is free.

The formal education system consists of four levels: pre-primary (2 years), primary (8 years), secondary (4 years), and tertiary (2+yrs Associate’s, 4+ Bachelor’s). Governed by the Education and Training Act of 2010, the organization of the Belizean education system falls within the Education Rules. These rules stipulate that the Ministry of Education, headed by a Minister of Education, collaborates with churches, communities, private, and other organizations to deliver education to its citizenry; however, local control and enforcement of policies is left to school management (Belize Management Information Systems, 2022).

The Belize Management Information System (2022) reports that education in Belize is compulsory from age 5–14. Education is primarily in person, with little to no online options or online schools. The education of students, whether at the primary or secondary level, largely takes place in public religious schools, predominantly Catholic schools. At the secondary and tertiary levels, there are more government-controlled schools. Primary school is equivalent to grades (1–8) in the United States while secondary is equivalent to high school (grades 9–12).

During the pandemic, a lot changed in Belize’s education—much like it did around the world. A COVID-19 oversight committee was formed; schools closed March 20, 2020, and went to remote teaching and learning after the Easter break (mid-April). Data collected in the national Belize Education Management Information system (BEMIS) continued, with certain changes. They collected no data on performance in math, English, social studies, and Science. They collected no attendance data for April, May, and June; and they did not record learning at home for pre-primary and primary (Flowers, 2020). According to a report to UNESCO (Flowers, 2020), records collection for students and teachers continued with pre-primary and primary school students receiving daily lessons and secondary and tertiary school students primarily being educated online. The report noted current gaps and difficulties in collecting data regarding absenteeism, school climate, support for teachers, primary and secondary school learning, and early childhood readiness to transition to primary school (Flowers, 2020). Additional areas the government of Belize hoped to collect data on and monitor were:

1. Ensuring students remain engaged and entertained without being overwhelmed.
2. Ensuring students without access to radio and television got paper copies of lessons.
3. Ensuring assessment of readiness for reopening and on track for learning.
4. Responding to limited student access to technology (devices and Internet service); and
5. Increasing the current low rate of parent education levels (many parents cannot help their children learn). (Flowers, 2020)

The sudden shift to online and distance learning created a change in lifestyles and introduced unique pandemic stressors for teachers, students, and parents. The teaching and learning process was now confined to the less interactive and more socially isolated home environments (Breaux et al., 2021; Lee et al., 2021; McElrath, 2020; Rosen et al., 2021; Scarpellini et al., 2021). The pivot to distance learning brought its own challenges for all involved (Gomez, 2020; Gomez et al., 2021). This was acutely so for students, resulting in an increase in stress, restlessness, boredom, loneliness, depression, aggressive behavior, and disruptions (Lee et al., 2021; Rosen et al., 2021; Scarpellini et al., 2021). Students’ competence, autonomy, and relatedness were also threatened through home confinement, school closure, and online learning. Specifically, students’ sense of autonomy in organizing and completing tasks at their own pace and on their own time without adequate support was threatened. Students’ sense of connection with others was also at risk as they no longer had any physical contact with their teachers and friends. The lack of satisfaction in competence, autonomy, and relatedness also hindered their academic motivation and engagement in the remote learning process (Zaccoletti et al., 2020).

Students in these less-than-ideal situations needed greater academic and social-emotional support to recover from the absence resulting from the pandemic. Prolonged time away from school, school absenteeism, has often been blamed for decreases in students’ academic behaviors (Balfanz & Byrnes, 2012; Fremont, 2003; Kearney et al., 2004; Wilkins, 2008). The pandemic offered an opportunity to study impact on academic behaviors and resilience, defined herein as *grit*. Eight factors are used to define academic behaviors: attendance, preparedness, attention, note-taking, participation, organization, use of out-of-school time, and homework completion and submission.

We define *grit* as the ability to show courage, passion, and perseverance in goal achievement or follow-through on tasks even under difficult circumstances (Duckworth, 2016; Duckworth & Quinn, 2009). This trait of resilience is not unidimensional (Luthar et al., 1993). It is a positive, noncognitive factor that aids in student motivation for academic and social success. Academic success, however, hinges not only on noncognitive factors but also on risk and protective factors, which can become risk chains given the strain put on the child by personal, familial, or school climate (Pollard et al., 1999; Smokowski et al., 2004).

Risk factors explored in this study rests on home and parental factors informed by socio-emotional health and levels of academic behaviors. Using the lens of ecological theory, we attempted to parse out the impact of a few risk factors that impede academic success because of absenteeism, specifically, their reciprocal effects on adolescents’ academic behaviors and grit. Ecological theory examines the multi-systems of influence (Fraser, 2004) and the simultaneous reciprocal relationships of people and their environment (Gitterman & Germain, 2008). In summary, the theory looks at the transactions and interactions between systems and their sub-systems (Bronfenbrenner & Morris, 2006).

We hypothesized that adolescents with disruption in or lower levels of the defined risk factors experienced more negative impact on their levels of academic behaviors and positive impact on grit during their absence from school during the pandemic.

## Review of the Related Literature

### COVID-19 and Effects on Education—Prolonged Absence

Around the world, education was disrupted during the COVID-19 pandemic. Learning behaviors changed as societies experienced changes to the way they functioned. Disruptions ranged from mask wearing, social/physical distancing, and school closings to remote/hybrid learning, vaccination and booster requirements, and weekly COVID-19 testing. Schools needed to respond to this disruption, which came at a cost for all stakeholders—administrators, teachers, parents, and students (Pozzoli et al., 2021; Scarpellini et al., 2021). In fact, it felt as if learning was being left to chance (Gross & Opalka, 2020). Starting with the abrupt school closure in March 2020, schools pivoted to emergency remote teaching, online/distance learning, and hybrid learning and eventually a return to in-person instruction on many campuses. All this was done to keep students learning though they were away from school for a prolonged period of time. Some students did not report or only partially reported to school, whether online or in-person. School absenteeism, a by-product of the pandemic, became pronounced. Many students, especially those from calm and stable homes, which were well (or more effectively) organized and supportive, adapted quickly to online and digital learning modes and thrived academically (Pozzoli et al., 2021). Other students had varied experiences, resulting sometimes in severe psychological effects (Brundin, 2021; Kidman et al., 2021; Scarpellini et al., 2021), such as stress, anxiety, and depression.

### School Absenteeism

In normal times, absenteeism data are not consistent because administrators, school districts, and researchers do not agree on what types of data to collect. *School absenteeism* (Gentle-Genitty, 2009; Keppens et al., 2019) and *school refusal* (Inglés et al., 2015) are terms used to describe school attendance problems (SAP). Each term has overly complex factors contributing to its continuance. Studies continue to reveal the typology of school attendance problems, ranging from school refusal and truancy to school withdrawal and exclusion (Heyne, Gren-Landell al., 2019), and common definitions are still being disentangled (Gentle-Genitty et al., 2015; Heyne, Gentle-Genitty et al., 2019). Regardless of the term used, Keppens and colleagues (2019) and Inglés and others (2015) concur that absenteeism is an issue that warrants attention especially since the associated problems are linked to lower academic performance, especially in math and English (Santibáñez & Guarino, 2021); increase in anxiety; symptoms of depression; poorer socio-emotional health; lower self-esteem; and socially deviant behaviors (substance abuse, vandalism, stealing) (Demir & Akman Karabeyoglu, 2015). During the pandemic, few risk factors had more of an obvious impact on students’ socio-emotional health than school closures and absenteeism. Around the globe, absenteeism was negatively correlated with students’ social awareness and self-efficacy (Patrick et al., 2020; Santibáñez & Guarino, 2021).

As this review of literature affirms, many factors, including absenteeism, home, socio-emotional, academic, and the like, impact the academic behaviors and grit of adolescents after prolonged school absence. Though these factors were present prior to the COVID-19 pandemic, their levels substantially increased and became more pronounced during the pandemic (Pozzoli et al., 2021; Scarpellini et al., 2021).

### Home and Parental Factors

When the COVID-19 pandemic started, we could not have predicted that school closures, job losses, parental death, and changes in parental marital structures would have deep impacts on absenteeism. The intervention strategies to respond to absenteeism may include all stakeholders from schools, parents, teachers, students, to the larger society (Inglés et al., 2015). It is because of this ecological structure of the problem that the second factor examined in this study concerns those related to the home or familial environment.

Regardless of the time spent in school, research has continued to confirm the long-term impact of factors related to home life and family on academic success (Bronfenbrenner & Morris, 2006; Pozzoli et al., 2021; Scarpellini et al., 2021). The home environment and the support students receive from their family members can positively or negatively affect students’ academic motivation and, ultimately, their academic success (Masten & Coatsworth, 1998; Masten et al., 2010). As research indicates, socioeconomic disadvantages severely impact childhood development (Hair et al., 2015; Jeon et al., 2014; McLoyd, 1998). A positive home climate is deemed fundamental to, and propulsive for, supporting successful learning. More specifically, children—younger children in particular—from homes that were calm, well organized, and supportive report less difficulty in learning especially in online classes (Pozzoli et al., 2021). The home environment changed during the pandemic, however. Both parents’ and students’ psychological well-being was at risk (Patrick et al., 2020). Households with school-aged children struggled with online learning and instructional time (McElrath, 2020) as well as technological self-efficacy (Pan, 2020). Consequently, the educational inequality gap is widely believed to have expanded during the COVID-19 pandemic (Pozzoli et al., 2021).

Scarpellini and colleagues (2021) noted that online learning, embodied in emergency remote teaching, lowered the quality of learning, and further suggested that prolonged remote learning may impact students’ future cognitive, emotional, and relational capacities. Students from lower socioeconomic homes were also at greater risk of not being able to access learning as they lacked the technological tools necessary. Students with a greater number of family socioeconomic risks and a higher level of neighborhood disadvantage demonstrated lower scores on cognitive skills and deficits in achievement (Jeon et al., 2014). The longer students live in poverty, the greater their academic deficits tend to be and, if allowed to persist to adulthood, these patterns contribute to lifetime-reduced occupational attainment (Hair et al., 2015; Masten et al., 2010) and may even affect the psychosocial development of offsprings in the next generation (Sameroff & Rosenblum, 2006). In other words, home learning environments can explain associations between family socioeconomic disadvantage and children’s cognitive skills and observable social-emotional problems (Jeon et al., 2014). This is crucial for Belize, as previous research on educational disparities and achievement gaps among students in Belize confirmed students from underserved communities are at greater risk of struggling academically and less likely to succeed in school and life (Palacio, 2013; Vairez et al., 2017).

According to Bronfenbrenner and Morris (2006), the stability or lack of stability of the family’s environment affects the social issues of children that may lead to violence and criminal behaviors. Proximal processes are not functional in-home environments that are unstable and unpredictable across space and time (Bronfenbrenner & Morris, 2006). Therefore, the presence of engaged parents in the lives of children helps to promote developmental activities and experiences. On the contrary, the absence of one parent in the lives of children can lead to lower academic performance, increased absenteeism from school, and other social issues. As such, the psychological trauma and bereavement students experienced from these cases of loss is further impetus to examine the impact of such noncognitive factors and behavior traits on students’ learning (Brundin, 2021). The psychological trauma factors are often defined as socio-emotional in nature.

### Academic Behaviors and Achievement

Academic performance is integral for student success, as discussed herein (Lee et al., 2021; Scarpellini et al., 2021; Zaccoletti et al., 2020). Such success is highly contingent on students being able to self-regulate their emotions in difficult times. Adequate emotional regulation strategies contribute to self-efficacious students who, in turn, have a positive outlook on learning and the classroom environment (Pozzoli et al., 2021). They also have a profound effect on students’ personal engagement and academic achievement (Boekaerts & Pekrun, 2016), which reflectively is evidenced in grade point averages (Gumora & Arsenio, 2002).

Academic behaviors have a direct effect on academic performance at all educational levels, from early childhood to high school and beyond (Farrington et al., 2012). Students earn better grades when they show perseverance and strong academic behaviors, reflected in engagement with school work. Students’ engagement in school work includes degree of attendance, completion of assignments on time, participation in class, studying, study skills (i.e., taking time to practice and learn their school work), and taking on challenging tasks and persevering until these tasks have been successfully completed (Farrington et al., 2012). In sum, students’ behaviors are shaped by their experiences in the classroom, their interactions with teachers and classmates, and their beliefs about their own abilities and the nature of the task at hand. There is a strong correlation with these influential external and internal factors and student success (Boekaerts & Pekrun, 2016; Farrington et al., 2012; Zaccoletti et al., 2020).

Evidence of the influential nature of these variables speaks to the role of the teacher. A study conducted by Pozzoli and colleagues (2021), with students ages 11 to 14 years from middle- and upper-class families, found that children who reported less difficulty with online classes included those students who perceived their teachers as showing more interest in their academic and psychological well-being. Pozzoli and colleagues (2021) suggest that teachers should be better trained to engage students in online learning as well as increase their closeness with students through the online context to improve students’ psychological well-being and, hence, their academic performance. Yet, during the COVID-19 pandemic and even prior, some teachers had concerns about techno-pedagogical self-efficacy and their own online teaching effectiveness (Cennamo et al., 2010; Gomez, 2020; Gomez et al., 2021). In fact, prior teacher competence and computer use led to fewer challenges in motivation and the e-learning environment for students because teachers (Fryer & Bovee, 2016) provided better support.

### Research Question and Purpose

As Scarpellini and colleagues (2021) suggest, distance/online learning in the form of emergency remote learning during the pandemic may not only have lowered the quality of learning but also adversely impacted students’ future cognitive, emotional, and relational capacities. This, in turn, may have widened the educational inequality gap stemming in part from family instability and/or absence of a parent.

Amidst the psychological trauma and bereavement students experienced from the COVID-19 pandemic, examination of their non-cognitive functioning and behavior traits on learning and development is necessary. Doing so leads to designing learning environments that foster students’ success, whether virtually or in person. In Belize, there was a call from the ministry of education for data on secondary school learning, early childhood readiness to transition to primary school, and impact of parental education levels (Flowers, 2020). Given prior relationships of the researchers with secondary and tertiary school partners, an online survey was prepared and administered to respond to the request for data.

A quantitative, causal-comparative study explored the effects of family status, parental marital status, family income, and parental education levels as risk factors on Belizean adolescents’ level of engagement in academic behaviors and grit during prolonged absence (Gentle-Genitty, 2009) due to the COVID-19 pandemic. These factors have been shown to increase students’ probability of being at risk for underachieving and failing academically or dropping out of school (Great Schools Partnership, 2013).

We conjectured that adolescents who experienced disruption in learning, or who possessed lower levels of the defined risk factors, and were out of school for a year or more due to COVID-19 school closings experienced more negative impact on academic behaviors and positive impact on grit. Eight academic behaviors were examined: attendance, preparedness, attention, note-taking, participation, organization, use of out-of-school time, and assignment completion and submission. For the purpose of the project, we defined *out-of-school time* as any time students spend outside the formal education system. This is often considered to range from 3:30pm through 8:00am and whole days during the weekend.

## Method

A quantitative research method with a causal-comparative research design was applied to explore the effects of family status, parental marital status, family income, and parental educational attainment on adolescents’ academic behaviors and grit following prolonged school absence due to COVID-19 in Belize. In a causal-comparative research design, the aim is to determine the cause, reason, or consequences of differences in behavior or status that exist between or among groups in a population (Fraenkel et al., 2012). That is, the goal is to determine the effects of the independent variable on the dependent variable by comparing two or more groups in a population (Salkind, 2010).

There are three types of causal-comparative research, focusing on exploration of effects, causes, and consequences (Fraenkel et al., 2012). This study focused on an exploration of effects. Causal-comparative research is referred to as ex-post facto (after the fact) research since the exploration is about conditions that already exist. Thus, in causal-comparative research, there is no manipulation of the independent variable, and random assignment to comparison groups is not possible (Fraenkel et al., 2012).

### Participants

The target population for this study was estimated to be 28,000 adolescents (ages 12–20 years) enrolled in high schools, junior colleges, and universities across the country of Belize. To establish the sample, adolescents enrolled in high schools were recruited through their teachers while adolescents from the junior colleges and universities were recruited through their instructors and directly via email to participate in the study. The intention was to give as many adolescents enrolled in high schools, junior colleges, and universities from across Belize an opportunity to participate in the study. The result was a sample consisting of 676 adolescents enrolled in high schools, junior colleges, and universities across Belize. Efforts were made to get a cross-sectional representation from government, government-aided, and private schools. The sample generated a 95% confidence level and 3.7% margin of error.

Of the 676 adolescents who participated in the study, 33.28% were males and 66.72% were females. Participating adolescents’ ages ranged from 12 to 20, with the mode age being 16 years old (21.89%). Majority of the adolescents (60.21%) were from urban areas. Further, most were from the Belize (42.31%) and Stann Creek districts (27.51%). Few adolescents were from the Cayo (10.80%), Corozal (7.40%), Toledo (6.21%), and Orange Walk (5.77%) districts. In terms of ethnicity, most adolescents self-identified as Mestizo (29.59%) and Creole (27.51%). Others self-identified as Garifuna (10.80%), multi-ethnic (10.80%), Maya (8.30%), and Hispanic/Central American (8.00%). Only a few adolescents self-identified as East Indian (2.51%), Mennonite (2.07%), and other (0.15%).

### Data Collection Procedures

Data were collected using an online instrument developed and administered to participants in the spring of 2021 via the Qualtrics online survey application. At that time, secondary and tertiary students would have been engaged primarily in remote learning starting in September of 2020. Students were still away from school. High school adolescents were recruited to participate in the study through their teachers. The link to the online survey with an invitation letter was emailed to high school teachers across Belize. The teachers then forwarded the link to the online survey with the invitation letter to their students. Adolescents who decided to participate in the study and were younger than 18 years had to complete an assent form and their parents had to complete a consent form before proceeding to complete the survey.

Adolescents enrolled in junior colleges and universities were recruited through their instructors, their peers, and directly via email to participate in the study. The link to the online survey with an invitation letter was emailed to adolescents enrolled in junior colleges and universities across Belize. Also, the link to the online survey with the invitation letter was also sent to the instructors from junior colleges and universities to forward to their students. Additionally, university students enrolled in an Adolescent Psychology course assisted with recruiting their peers and their students to participate in the study. The online survey was opened for two months to complete the data collection phase of the project. During this period, weekly reminders were sent out to recruiters and participants to complete the survey. Language barriers and translation were not identified as challenges for the students. Therefore, translation services or assistance was not needed nor provided.

### Measures

The online survey used to collect the data for this study consisted of three sections. The independent variables were family status, parental marital status, family income, and parental educational attainment. These variables were measured via Section 1, the demographic profile section of the online survey, which required participants to select the appropriate category for each item. The variable family status was measured on a nominal scale with two categories: single-parent family and nuclear family. The presence of two parents in the home was classified as a nuclear family regardless of formal status. Common unions are recognized by Belize statutes and codes as legally binding. In fact, according to the 2017 case of *Flowers vs. Jeffords*, a common law union in Belize is defined as an “unmarried man and an unmarried woman who share a mutual commitment publicly to live their life together as a couple and in fact do so for a continuous period of five years or more” (Belize Judiciary, 2018; Legal Information Institute, n.d.).

The variable parental marital status was measured on a nominal scale with four categories: single, married, divorced/separated, and widowed. The family income variable was measured on an ordinal scale with 11 levels (1 = less than $100 to 11 = more than $1,000). Later, this variable was transformed to a dichotomous variable where 1 = weekly income less than BZ$200 (US$100) and 2 = weekly income greater than BZ$200. This financial benchmark was selected based on the per-capita income for the country of Belize. On average, the per-capita income was US$4,906 in 2016 and US$4,806.50 in 2017, resulting in approximately BZ$200 per week (Doby, 2018; Lano, 2017).

Finally, the variable parents’ educational attainment was measured on an ordinal scale with 8 levels (1 = some primary schools to 8 = doctorate degree). Later, this variable was transformed to a dichotomous variable where 1 = no college and 2 = college degree.

The dependent variables were adolescents’ grit and academic behaviors. Section 2 included the Grit-S Scale, which is an efficient measure of grit—perseverance and passion for long-term goals (Duckworth & Quinn, 2009). The Grit-S Scale has eight items using a 5-point Likert-type scale (1 = Not like me at all, 2 = Not much like me, 3 = Somewhat like me, 4 = Mostly like me and 5 = Very much like me). Adolescents’ grit scores were computed by reverse coding of Items 1, 3, 5 and 6 and then adding up all the points for the eight items and dividing by eight to establish a composite/index grit score. The maximum score on this scale is 5 (extremely gritty); the lowest is 1 (not at all gritty) (Duckworth & Quinn, 2009). The Grit-S Scale has strong psychometric properties with evidence for predictive validity, consensual validity, and test-retest stability and acceptable internal consistency, with Cronbach’s alphas between .73 to .83 (Duckworth & Quinn, 2009).

Finally, Section 3 addressed the second dependent variable, academic behaviors. It consisted of items related to the eight target academic behaviors based on Farrington and colleague’s (2012) framework of noncognitive factors. The eight items used the same 5-point Likert-type scale (1 = Not like me at all, 2 = Not much like me, 3 = Somewhat like me, 4 = Mostly like me and 5 = Very much like me) as the Grit-S Scale. Examples of items include: I attend *class regularly; I arrive to class prepared and ready to work; and I pay attention in class* (see Table 1). Since these items did not measure a single dimension or construct, a composite score was not computed. Instead, each item (behavior) was analyzed and reported individually.

**Table 1:** Academic Behaviors and Grit by Family Status.


	FAMILY STATUS	*N*	*M*	*SD*

**AB — 1. I attend class regularly.	Single-Parent Family	310	4.08	1.269

	Nuclear Family	299	4.32	1.216

AB — 2. I arrive to class prepared and ready to work.	Single-Parent Family	310	3.80	1.203

	Nuclear Family	299	3.89	1.206

AB — 3. I pay attention in class.	Single-Parent Family	310	3.65	1.129

	Nuclear Family	299	3.77	1.112

AB — 4. I take notes in class.	Single-Parent Family	310	3.46	1.352

	Nuclear Family	299	3.48	1.317

AB — 5. I participate in instructional activities and class discussions.	Single-Parent Family	310	3.38	1.296

	Nuclear Family	299	3.47	1.254

AB — 6. I keep my school books and materials organized.	Single Parent Family	310	3.63	1.303

	Nuclear Family	299	3.65	1.334

*AB — 7. I devote out-of-school time to studying.	Single-Parent Family	310	2.97	1.232

	Nuclear Family	299	3.15	1.241

*AB — 8. I complete and submit my homework on time.	Single-Parent Family	310	3.71	1.247

	Nuclear Family	299	3.91	1.309

Grit Score	Single-Parent Family	310	3.26	.661

	Nuclear Family	299	3.27	.611


* *p* < .10. ** *p* < .05.

### Data Analysis

To explore the effects of the risk factors (family status, parental marital status, family income, and parent education level) on adolescents’ academic behaviors and grit, we analyzed the data by computing descriptive and inferential statistics using IBM SPSS version 23 statistical software. First, we computed measures of central tendency and dispersion. Subsequently, we used independent samples *t*-tests and one-way analysis of variance (one-way ANOVA) to test four null hypotheses. Thus, four statistical analyses were conducted.

## Results

The first analysis was conducted to determine the effect of adolescents’ family status on their academic behaviors and grit. The null hypothesis tested asserted that there were no significant differences in adolescents’ academic behaviors and grit mean scores between adolescents from single-parent and nuclear families. Nine independent samples *t*-tests were conducted to test this hypothesis.

The results revealed adolescents from single-parent families, on average, had lower levels of academic behaviors than their peers from nuclear families across the eight academic behaviors explored (see Table 1). Of the eight academic behaviors, three showed significant differences in mean scores: *I attend class regularly* [*t* (607) = 2.320, *p* = .021]*, I devote out-of-school time to studying* [*t* (607) = 1.825, *p* = .068], and *I complete and submit my homework on time* [*t* (607) = 1.963, *p* = .050]. There was no significant difference in mean grit scores between adolescents from single-parent and those from nuclear families.

The second analysis was conducted to determine the effect of parental marital status on adolescents’ academic behaviors and grit. The null hypothesis asserted that there were no significant differences in adolescents’ academic behaviors and grit mean scores regardless of parental marital status (single, married, divorced/separated, and widowed parents). Nine one-way ANOVA tests were used to test this hypothesis.

The results showed that five of the nine one-way ANOVA tests were significant: *I attend class regularly* [*F*(3, 605) = 2.495, *p* = .059], *I pay attention in class* [*F*(3, 605) = 2.646, *p* = .048], *I participate in instructional activities and class discussions* [*F*(3, 605) = 2.720, *p* = .044], *I keep my school books and materials organized* [*F*(3, 605) = 2.132, *p* = .095], and *I complete and submit my homework on time* [*F*(3, 605) = 2.638, *p* = .049]. Further, adolescents who experienced the psychological trauma of divorce or death of a parent had lower mean scores on the eight academic behaviors and grit levels than their peers whose parents were single or married (see Table 2).

**Table 2:** Academic Behaviors and Grit Descriptive Statistics by Parents’ Marital Status.


	PARENTS’ MARITAL STATUS	*N*	*M*	*SD*

*AB — 1. I attend class regularly.	Single	154	4.08	1.266

	Married	299	4.32	1.216

	Divorced/Separated	131	4.15	1.243

	Widowed	25	3.76	1.422

AB — 2. I arrive to class prepared and ready to work.	Single	154	3.90	1.181

	Married	299	3.89	1.206

	Divorced/Separated	131	3.74	1.213

	Widowed	25	3.52	1.262

**AB — 3. I pay attention in class.	Single	154	3.69	1.157

	Married	299	3.77	1.112

	Divorced/Separated	131	3.71	1.063

	Widowed	25	3.12	1.201

AB — 4. I take notes in class.	Single	154	3.58	1.347

	Married	299	3.48	1.317

	Divorced/Separated	131	3.42	1.358

	Widowed	25	2.92	1.256

**AB — 5. I participate in instructional activities and class discussions.	Single	154	3.42	1.272

	Married	299	3.47	1.254

	Divorced/Separated	131	3.45	1.272

	Widowed	25	2.72	1.429

*AB — 6. I keep my school books and materials organized.	Single	154	3.75	1.296

	Married	299	3.65	1.334

	Divorced/Separated	131	3.60	1.264

	Widowed	25	3.04	1.428

AB — 7. I devote out-of-school time to studying.	Single	154	3.08	1.255

	Married	299	3.15	1.241

	Divorced/Separated	131	2.83	1.235

	Widowed	25	3.04	1.020

**AB — 8. I complete and submit my homework on time.	Single	154	3.79	1.219

	Married	299	3.91	1.309

	Divorced/Separated	131	3.69	1.221

	Widowed	25	3.24	1.480

Grit Score	Single	154	3.27	.695

	Married	299	3.27	.611

	Divorced/Separated	131	3.29	.621

	Widowed	25	3.10	.651


* *p* < .10. ** *p* < .05.

Subsequently, post hoc multiple comparisons using Tukey’s honest significant difference (HSD) tests were conducted on the five academic behaviors with significant differences to ascertain which pairs of subgroups of adolescents had significantly different mean scores. Doing so allowed affirmation of the effect of the risk factor, parental marital status, on adolescents’ academic behaviors and grit.

The results revealed that the mean score for the academic behavior of *I pay attention in class* for adolescents from widowed parents (*M* = 3.12, *SD* = 1.201) was significantly lower than for adolescents of single (*M* = 3.69, *SD* = 1.157, *p* = .081), married (*M* = 3.77, *SD* = 1.112, *p* = .027), and divorced/separated parents (*M* = 3.71, *SD* = 1.063, *p* = .074). Likewise, the mean score of *I participate in instructional activities and class discussions* for adolescents of widowed parents (*M* = 2.72, *SD* = 1.429) was significantly lower than for adolescents of single (*M* = 3.42, *SD* = 1.272, *p* = .052), married (*M* = 3.47, *SD* = 1.254, *p* = .024), and divorced/separated parents (*M* = 3.45, *SD* = 1.272, *p* = .043).

Finally, the Tukey’s HSD tests also showed that the mean score for the academic behavior of *I keep my school books and materials organized* for adolescents of widowed parents (*M* = 3.04, *SD* = 1.428) was significantly lower than for adolescents of single parents (*M* = 3.75, *SD* = 1.296, *p* = .062). The mean score for the academic behavior of *I devote out-of-school time to studying* for adolescents of divorced/separated parents (*M* = 2.83, *SD* = 1.235) was significantly lower than for adolescents of married parents (*M* = 3.15, *SD* = 1.241, *p* = .063). Lastly, the mean score for the academic behavior of *I complete and submit my homework on time* for adolescents of widowed parents (*M* = 3.24, *SD* = 1.480) was significantly lower than for adolescents of married parents (*M* = 3.91, *SD* = 1.309, *p* = .058).

In sum, these results indicate adolescents of widowed parents, on average, had lower scores than peers on both academic behaviors and grit levels. In other words, living through the COVID-19 pandemic and being absent from school for a prolonged period had a more adverse effect on adolescents who experienced the psychological trauma of the death of a parent. Accordingly, these adolescents consistently had significantly lower mean scores on most of the eight academic behaviors explored than their peers of single and married parents.

The third analysis was conducted to determine the effect of adolescents’ family income on their academic behaviors and grit. The null hypothesis tested asserted that there were no significant differences in academic behaviors and grit mean scores between adolescents from families with weekly income less than BZ$200 (US$100) and those from families with weekly income greater than BZ$200. This financial benchmark was selected based on the per-capita income for Belize. On average, the per capita was US$4,906 in 2016 and US$4,806.50 in 2017, resulting in approximately BZ$200 per week (Doby, 2018; Lano, 2017).

To test this hypothesis, nine independent samples *t*-tests were performed. The results indicated adolescents from families with weekly income greater than BZ$200, on average, had lower levels of academic behaviors than their peers from families with weekly income less than BZ$200 for seven of the eight academic behaviors explored (see Table 3). However, of the eight academic behaviors, the differences in mean scores were only significant for three academic behaviors: *I arrive to class prepared and ready to work* [*t* (607) = 1.891, *p* = .059]*, I take notes in class* [*t* (607) = 3.025, *p* = .003], and *I keep my school books and materials organized* [*t* (607) = 2.024, *p* = .043]. This suggests adolescents with lower income-earning parents were more prepared, ready to learn in class, take class notes, and were organized with the materials and resources available to them during the COVID-19 pandemic than adolescents with higher income-earning parents.

**Table 3:** Academic Behaviors and Grit Descriptive Statistics by Parents’ Weekly Income.


	PARENTS’ WEEKLY INCOME	*N*	*M*	*SD*

AB — 1. I attend class regularly.	<$200	129	4.28	1.139

	>$200	480	4.18	1.276

*AB — 2. I arrive to class prepared and ready to work.	<$200	129	4.02	1.027

	>$200	480	3.80	1.244

AB — 3. I pay attention in class.	<$200	129	3.79	1.066

	>$200	480	3.69	1.136

**AB — 4. I take notes in class.	<$200	129	3.78	1.281

	>$200	480	3.39	1.337

AB — 5. I participate in instructional activities and class discussions.	<$200	129	3.50	1.238

	>$200	480	3.40	1.286

**AB — 6. I keep my school books and materials organized.	<$200	129	3.84	1.326

	>$200	480	3.58	1.311

AB — 7. I devote out-of-school time to studying.	<$200	129	3.17	1.140

	>$200	480	3.03	1.263

AB — 8. I complete and submit my homework on time.	<$200	129	3.81	1.219

	>$200	480	3.81	1.298

Grit Score	<$200	129	3.19	.652

	>$200	480	3.29	.632


* *p* < .10. ** *p* < .05. *Note*: All currency cited is in Belize (BZ) dollars, which has approximately a 2-to-1 exchange rate with the US dollar.

In contrast, the results indicated adolescents from families with weekly incomes greater than BZ$200, on average, had higher levels of grit than their peers from families with weekly incomes less than BZ$200 (see Table 3). However, the difference in mean grit scores between adolescents from families with weekly incomes greater than BZ$200 and their peers from families with weekly incomes less than BZ$200 was not significant. This implies parents’ financial situation and standard of living afforded to adolescents, on average, did not significantly impact their grit during the COVID-19 pandemic while being absent from school for a prolonged period.

The final analysis was conducted to determine the effect of adolescents’ parental educational attainment on their academic behaviors and grit. The null hypothesis tested asserted there were no significant differences in academic behaviors and grit mean scores between adolescents with parents who had no college education and those with parents who had a college education. To test this hypothesis, another nine independent samples *t*-tests were conducted.

The results showed adolescents with parents who had no college education, on average, had higher levels of academic behaviors than their peers with parents who had a college education for all eight academic behaviors explored (see Table 4). Notably, of the eight academic behaviors, the differences in mean scores were significant for five: *I arrive to class prepared and ready to work* [*t* (607) = 1.824, *p* = .069]*, I pay attention in class* [*t* (607) = 2.383, *p* = .017]*, I take notes in class* [*t* (607) = 1.997, *p* =.046], *I keep my school books and materials organized* [*t* (607) = 2.449, *p* = .015], and *I complete and submit my homework on time* [*t* (607) = 2.788, *p* = .005]. These results suggest adolescents with parents who had no college education were challenged by their circumstances to become self-directed, develop good work and study habits, and advance their organizational and executive functioning skills.

**Table 4:** Academic Behaviors and Grit Descriptive Statistics by Parents’ Educational Attainment.


	PARENTS’ ED. ATTAINMENT	*N*	*M*	*SD*

AB — 1. I attend class regularly.	No College	366	4.24	1.186

	College Degree	243	4.13	1.336

*AB — 2. I arrive to class prepared and ready to work.	No College	366	3.92	1.158

	College Degree	243	3.74	1.265

**AB — 3. I pay attention in class.	No College	366	3.80	1.111

	College Degree	243	3.58	1.127

**AB — 4. I take notes in class.	No College	366	3.56	1.363

	College Degree	243	3.34	1.280

AB — 5. I participate in instructional activities and class discussions.	No College	366	3.46	1.289

	College Degree	243	3.37	1.255

**AB — 6. I keep my school books and materials organized.	No College	366	3.74	1.314

	College Degree	243	3.48	1.309

AB — 7. I devote out-of-school time to studying.	No College	366	3.13	1.252

	College Degree	243	2.96	1.214

**AB — 8. I complete and submit my homework on time.	No College	366	3.92	1.212

	College Degree	243	3.63	1.362

*Grit Score	No College	366	3.30	.644

	College Degree	243	3.21	.622


* *p* < .10. ** *p* < .05.

Likewise, the results indicated adolescents with parents who had no college education, on average, had higher levels of grit than their peers with parents who had a college education (see Table 4). The difference in mean grit scores between adolescents with parents who had no college education and their peers with parents who had a college education was significant [*t* (607) = 1.714, *p* = .087]. These results suggest that during prolonged absence from school due to the COVID-19 pandemic, adolescents with parents who had no college education manifested better academic behaviors and higher levels of perseverance and passion for long-term goals than their peers whose parents had a college education.

## Discussion

This quantitative causal-comparative study resulted in several important findings and implications for fostering and developing adolescents’ academic behaviors and grit. All factors examined—attendance, preparation, attention, note-taking, participation, organization, out-of-school time to study, complete and submit work and grit—are foundational to success in school and life (Farrington et al., 2012).

Our key findings suggest adolescents from single-parent families had lower levels of academic behaviors than their peers from nuclear families across the eight behaviors explored. This effect was more severe for adolescents who experienced the psychological trauma of divorce or death of a parent. Adolescents from widowed parents, on average, had lower scores than their peers on academic behaviors and grit. The COVID-19 pandemic and being absent from school for a prolonged period had a more adverse effect on adolescents who experienced the psychological trauma of the death of a parent (Kidman et al., 2021). Accordingly, these adolescents consistently had significantly lower mean scores for the five academic behaviors with significant differences as compared to their peers from single- and married-parents families.

Further, the results indicated adolescents from nuclear and higher income families had slightly higher levels of grit. This is attributable to the fact that parents in nuclear families and those with higher income earnings are presumably more likely to provide greater support to the adolescents in their families with time, mentoring, tutoring and homework supervision, adequate supplies, and resource materials. These adolescents may also have more access to such tools as technology, reliable Internet connectivity, and digital devices like iPads, laptops, and desktop computers. Adolescents of parents with lower levels of educational attainment had higher levels of academic behaviors and grit. In the context of this study, during prolonged absence from school due to the COVID-19 pandemic, adolescents with parents who had no college education manifested better academic behaviors and higher levels of perseverance and passion for long-term goals than their peers whose parents had a college education.

These implications may speak to under-resourced adolescents having to develop self-discipline and self-directedness, good work and study habits, organizational and executive functioning skills early in life. These attitudes of persistence, passion, and determination may have helped to overcome their parents’ limited support and assistance because of education level or formal education (Masten et al., 1999; Sameroff & Rosenblum, 2006). Presumably, this gritty mindset of resilience may also have resulted from parental efforts to instill the value of obtaining a good education to improve future financial empowerment and social mobility (Masten & Coatsworth, 1998; Masten et al., 2010). Concomitantly, the adaptation and development of positive noncognitive behavior traits may also be the adolescents’ impetus to escape poverty and life’s hardships stemming from their communal and familial experiences of living with parents lacking a college education.

Nonetheless, to meet all students’ needs, student engagement and motivation in the classroom need to be supported on and offline (Fryer & Bovee, 2016). In turn, for students’ motivation deficits to be a priority to alleviate academic challenges, teachers’ well-being, workload, and support matter, both off and online (Fryer & Bovee, 2016). Since teachers can address a broad array of motivational deficits, it is important for school officials and parents to support them as students who start with critical deficits in motivation generally further degrade over time (Fryer & Bovee, 2016; Fryer et al., 2014).

## Limitations

This study is not without its limitations. Though the sample size was large, the study was conducted in one country with a multi-ethnic and multicultural population, which may affect generalizability. Though many restrictions have been lifted, the pandemic is still not over (or has not been declared over in Belize). More data are being compiled daily; therefore, the reported finding may change and the ultimate impact on adolescents may remain unclear. The impact of prolonged absenteeism and influence of noncognitive factors on adolescents’ academic behaviors, grit, and achievement is presumably subject to change. Therefore, the findings from this study should be taken with that caveat in mind.

The results offer a snapshot in time serving as descriptive reminders about the need for prescriptive measures to meet the needs of students and improve the quality of instruction and educational services. The pandemic lingers and continues to be psychologically draining on both students and educators; we must foster learning environments that support both.

Also, this study explored the effects of four major risk factors (family status, parental marital status, family income, and parent educational attainment) on adolescents’ academic behaviors and grit. However, other factors (such as students’ second-language acquisition, poor school attitude or deficits in motivation, low ability level or prior achievement gaps, low self-esteem/self-efficacy as well as low parental availability, support, and expectations) may have exerted an influence on the risk factors investigated. They were outside the scope of this study and could be explored in future studies.

A final limitation speaks to the self-reported nature of the data, which plays a role in the interpretation of the findings and the consideration afforded to the external validity and future directions of this research. These concerns of limitation merit further investigation to foster greater corroboration of the findings from this study. However, we hope that any such replications or extensions would be based on a similar size or larger sample and employ a causal-comparative research design to ensure meaningful comparison with the current study’s findings.

## Conclusions and Recommendations

The support and availability of parents—single, married, divorced/separated, or widowed—and their financial situation and standard of living (as afforded by their education levels) have varying degrees of impact on adolescents’ academic behaviors and grit. This may be singly or in tandem, especially when there is prolonged absence from school due to the COVID-19 pandemic. Regardless, it requires changing the way we respond to students who have been absent (Gentle-Genitty et al., 2020). Student support matters, both off and online (Fryer & Bovee, 2016). This is especially important in remote learning environments, where students who start with critical deficits in motivation generally further degrade over time (Fryer et al., 2014).

We recommend schools become trauma responsive in support of adolescents who experience death or divorce of a parent and other traumatic experiences due to the prolonged absence from school and in-person instruction caused by the disruption of the COVID-19 pandemic. Trauma response requires engagement of multiple targets inclusive of care, sensitivity, cultural change, voice and choice, safety, and resources (Fondren et al., 2020). The COVID-19 pandemic took a heavy toll on students’ mental health. For example, students experienced increases in anxiety, sluggish cognitive tempo (SCT), inattention, and oppositionality/defiance symptoms, attention deficit hyperactivity disorder (ADHD) and poor emotional regulation abilities, resulting in higher externalizing behaviors (Breaux et al., 2021).

A significant number of students during the pandemic had added pressures due to their inability to access technological tools (McElrath, 2020) leading to a decrease in academic motivation because of prolonged absence from school. Academic motivation is a predictor of factors that might negatively or positively affect students’ trajectories in schools. Students who are highly motivated are more likely to excel in school, including in extracurricular activities (Camacho et al., 2021; Zaccoletti et al., 2020). The development of adequate emotional regulation skills and high-quality instruction are good strategies to help enhance students’ academic achievement (Boekaerts & Pekrun, 2016; Sameroff & Rosenblum, 2006).

Educators may need to be supportive of all adolescents regardless of their parental educational attainment or income level. Such changes in school climate may foster the development of better academic behaviors, grit, and psychological well-being. In doing so, attention is also given to attendance and the need for communication with all stakeholders—from school administrators, parents, teachers, students, to the larger society—for student success (Heyne, Gentle-Genitty et al., 2019).

In short, adolescents’ level of academic behaviors and grit should be constantly assessed to design learning environments that foster adolescents’ success in school and later in life. Failure to do so will compound the existing educational disparities in Belize among students from underserved communities, which is detrimental to the future of Belize (Palacio, 2013; Vairez et al., 2017).

We would be remiss to not also suggest that future studies be carried out in other countries to explore other school factors that affect adolescents such as modality of learning, type of school, location of school, access to resources, and so on, to assess and compare such factors during and after the COVID-19 pandemic. Similarly, future studies could further disaggregate adolescent groups—such as high-schoolers vs. college students; males vs. females; urban vs. rural; as well as comparative analyses at the district and regional levels, and so on—to investigate how academic behaviors and grit are impacted following prolonged absence due to the COVID-19 pandemic.

Finally, it is incumbent upon us to suggest that the concerns of limitation call for further investigation to foster greater corroboration of the findings from this study and, more important, to better meet the present and future educational needs of Belize’s growing and promising adolescent population. Therefore, replication or extension of this study is welcomed. Of note, in future studies there is the need to expand participation to be inclusive of a larger portion of adolescents from rural areas outside of Belize City to increase representativeness and generalizability.

## References

[1] Balfanz, R., & Byrnes, V. (2012). The importance of being in school: A report on absenteeism in the nation’s public schools. Retrieved from http://new.every1graduates.org/wp-content/uploads/2012/05/finalchronicabsenteeismreport_may16.pdf

[2] Belize Judiciary. (2018). Supreme Court claim no. 452 of 2017 Delsie Flowers v. Alton Jeffords. Retrieved from http://belizejudiciary.org/wp-content/uploads/2018/01/Supreme-Court-Claim-No-452-of-2017-Delsie-Flowers-v-Alton-Jeffords.pdf

[3] Belize Management Information Systems. (2022). Education system overview [A Government of Belize Website]. Belize: Ministry of Education, Culture, Science and Technology. Retrieved from https://www.moecst.gov.bz/education-sectors/education-system-overview/

[4] Belize World Factbook. (2021). Explore all countries: Belize, Central America. Retrieved from https://www.cia.gov/the-world-factbook/countries/belize/

[5] Boekaerts, M., & Pekrun, R. (2016). Emotions and emotion regulation in academic settings. In L. Corno & E. M. Anderman (Eds.), Handbook of educational psychology (pp. 76–90). Routledge/Taylor & Francis Group.

[6] Breaux, R., Dvorsky, M. R., Marsh, N. P., Green, C. D., Cash, A. R., Shroff, D. M., Buchen, N., Langberg, J. M., & Becker, S. P. (2021). Prospective impact of COVID-19 on mental health functioning in adolescents with and without ADHD: Protective role of emotion regulation abilities. Journal of Child Psychology and Psychiatry, 62(9), 1132–1139. DOI: 10.1111/jcpp.13382 33543486 PMC8014657

[7] Bronfenbrenner, U., & Morris, P. A. (2006). The bioecological model of human development. In R. M. Lerner & W. Damon (Eds.), Handbook of child psychology: Vol. 1. Theoretical models of human development (6th ed., pp. 793–828). John Wiley & Sons Inc.

[8] Brundin, J. (2021, April 5). All kinds of trauma: Students are returning to school, but are we ready to help them cope? Colorado Public Radio News. Retrieved from https://www.cpr.org/2021/04/05/all-kinds-of-trauma-students-are-returning-to-school-but-are-we-ready-to-help-them-cope/

[9] Camacho, A., Correia, N., Zaccoletti, S., & Daniel, J. R. (2021). Anxiety and social support as predictors of student academic motivation during the COVID-19. Frontiers in Psychology, 12(644338), 1–11. DOI: 10.3389/fpsyg.2021.644338 PMC8183683 34108910

[10] Cennamo, K. S., Ross, J. D., & Ertmer, P. A. (2010). Technology integration for meaningful classroom use: A standards-based approach. Wadsworth Cengage Learning.

[11] Demir, K., & Akman Karabeyoglu, Y. (2015). Factors associated with absenteeism in high schools. Eurasian Journal of Educational Research, 62, 37–56. Retrieved from https://files.eric.ed.gov/fulltext/EJ1097992.pdf. DOI: 10.14689/ejer.2016.62.4

[12] Doby, L. (2018). Path to improvement: Top 10 facts about poverty in Belize [Blog]. The Borgen Project. Retrieved from https://borgenproject.org/path-to-improvement-top-10-facts-about-poverty-in-belize/

[13] Duckworth, A. (2016). Grit: The power of passion and perseverance. Scribner Book Company.

[14] Duckworth, A. L., & Quinn, P. D. (2009). Development and validation of the Short Grit Scale (Grit-S). Journal of Personality Assessment, 91(2), 166–174. DOI: 10.1080/00223890802634290 19205937

[15] Farrington, C. A., Roderick, M., Allensworth, E., Nagaoka, J., Keyes, T. S., Johnson, D. W., & Beechum, N. O. (2012). Teaching adolescents to become learners. The role of noncognitive factors in shaping school performance: A critical literature review. University of Chicago Consortium on Chicago School Research.

[16] Flowers, Y. C. (2020). COVID-19 and education in Belize [Presented to UNESCO]. Retrieved from https://tcg.uis.unesco.org/wp-content/uploads/sites/4/2020/05/UIS_COVID_Belize.pdf

[17] Fondren, K., Lawson, M., Speidel, R., McDonnell, C. G., & Valentino, K. (2020). Buffering the effects of childhood trauma within the school setting: A systematic review of trauma-informed and trauma-responsive interventions among trauma-affected youth. Children and Youth Services Review, 109(11), 104691. DOI: 10.1016/j.childyouth.2019.104691

[18] Fraenkel, J., Wallen, N., & Hyun, H. (2012). How to design and evaluate research in education. McGraw-Hill, Inc.

[19] Fraser, M. (Ed.). (2004). Risk and resilience in childhood: An ecological perspective. NASW Press.

[20] Fremont, W. (2003). School refusal in children and adolescents. American Family Physician, 68(8), 1555–1560. Retrieved from https://aafp.org/afp/2003/1015/p1555.html 14596443

[21] Fryer, L. K., & Bovee, H. N. (2016). Supporting students’ motivation for e-learning: Teachers matter on and off line. The Internet and Higher Education, 30, 21–29. DOI: 10.1016/j.iheduc.2016.03.003

[22] Fryer, L. K., Bovee, H. N., & Nakao, K. (2014). E-learning: Reasons students in language learning courses do not want to. Computers & Education, 74, 26–36. DOI: 10.1016/j.compedu.2014.01.008

[23] Gentle-Genitty, C. (2009). Tracking more than absences: Impact of school’s social bonding on chronic truancy. Lambert Academic Publishing. DOI: 10.1037/e625252012-001

[24] Gentle-Genitty, C., Karikari, I., Chen, H., Wilka, E., & Kim, J. (2015). Truancy: A look at definitions in the USA and other territories. Educational Studies, 41(1–2), 62–90. DOI: 10.1080/03055698.2014.955734

[25] Gentle-Genitty, C., Taylor, J., & Renguette, C. (2020). A change in the frame: From absenteeism to attendance. Frontiers in Education, 4(161), 1–6. DOI: 10.3389/feduc.2019.00161

[26] Gitterman, A., & Germain, C. B. (2008). Ecological framework (e-reference edition). In T. Mizrahi & L. E. Davis (Eds.), Encyclopedia of social work. National Association of Social Workers and Oxford University Press, Inc. DOI: http://www.oxford-naswsocialwork.com/entry?entry=t203.e118

[27] Gomez, F. C., Jr. (2020). Technology integration self-efficacy reframed through the ISTE standards: An investigation among urban K-12 teachers [Doctoral dissertation, Boise State University, Boise, ID]. Retrieved from https://scholarworks.boisestate.edu/td/1692/

[28] Gomez, F. C., Jr., Trespalacios, J., Hsu, Y., & Yang, D. (2021). Exploring teachers’ integration self-efficacy through the 2017 ISTE standards. TechTrends. DOI: 10.1007/s11528-021-00639-z PMC8339164 34378007

[29] Great Schools Partnership. (2013). At-risk. In Glossary of education reform. Retrieved from https://www.edglossary.org/at-risk/

[30] Gross, B., & Opalka, A. (2020). Too many schools leave learning to chance during the pandemic. Center on Reinventing Public Education. Retrieved from https://crpe.org/too-many-schools-leave-learning-to-chance-during-the-pandemic/

[31] Gumora, G., & Arsenio, W. F. (2002). Emotionality, emotion regulation, and school performance in middle school children. Journal of School Psychology, 40(5), 395–413. DOI: 10.1016/S0022-4405(02)00108-5

[32] Hair, N. L., Hanson, J. L., Wolfe, B. L., & Pollak, S. D. (2015). Association of child poverty, brain development, and academic achievement. AMA Pediatrics, 169(9), 822–829. DOI: 10.1001/jamapediatrics.2015.1475 PMC4687959 26192216

[33] Heyne, D., Gentle-Genitty, C., Gren-Landell, M. G., Melvin, G., Chu, B., Galle-Tessonneau, M., Gärtner Askeland, K., Gonzálvez, C., Havik, T., Magne Ingul, J., Bach Johnsen, D., Keppens, G., Knollmann, M., Lyon, A. R., Maeda, N., Reissner, V., Sauter, F., Silverman, W. K., Thastum, M., Tonge, B. J., & Kearney, C. A. (2019). Improving school attendance by enhancing communication among stakeholders: Establishment of the International Network for School Attendance (INSA). European Child and Adolescent Psychiatry, 365(105585), 18. DOI: 10.1007/s00787-019-01380-y 31372748

[34] Heyne, D., Gren-Landell, M., Melvin, G., & Gentle-Genitty, C. (2019). Differentiation between school attendance problems: Why and how? Cognitive and Behavioral Practice, 26(1), 8–34. DOI: 10.1016/j.cbpra.2018.03.006

[35] Human Development Index (HDI). (2020). Human development reports. Retrieved from http://hdr.undp.org/en/content/human-development-index-hdi

[36] Inglés, C. J., Gonzálvez-Maciá, C., García-Fernández, J. M., Vicent, M., & Martínez-Monteagudo, M. C. (2015). Current status of research on school refusal. European Journal of Education and Psychology, 8(1), 37–52. DOI: 10.1016/j.ejeps.2015.10.005

[37] Jeon, L., Buettner, C. K., & Hur, E. (2014). Family and neighborhood disadvantage, home environment, and children’s school readiness. Journal of Family Psychology, 28(5), 718–727. DOI: 10.1037/fam0000022 25150370

[38] Kearney, C. A., Lemos, A., & Silverman, J. R. (2004). The functional assessment of school refusal behavior. The Behavior Analyst Today, 5(3), 275–283. Retrieved from https://eric.ed.gov/?id=ej1072107. DOI: 10.1037/h0100040

[39] Keppens, G., Spruyt, B., & Dockx, J. (2019). Measuring school absenteeism: Administrative attendance data collected by schools differ from self-reports in systematic ways. Frontiers in Psychology, 10(2623), 1–10. DOI: 10.3389/fpsyg.2019.02623 31849752 PMC6901495

[40] Kidman, R., Margolis, R., & Smith-Greenaway, E. (2021). Estimates and projections of COVID-19 and parental death in the US. JAMA Pediatrics, 175(7), 745–746. Retrieved from https://jamanetwork.com/journals/jamapediatrics/fullarticle/2778229/33818598 10.1001/jamapediatrics.2021.0161 PMC8022263

[41] Lano, C. (2017). Economic context for poverty in Belize [Blog]. The Borgen Project. Retrieved from https://borgenproject.org/poverty-in-belize-2/.

[42] Lee, S. J., Ward, K. P., Chang, O. D., & Downing, K. M. (2021). Parenting activities and the transition to home-based education during the COVID-19 pandemic. Children and Youth Services Review, 122(105585), 1–10. DOI: 10.1016/j.childyouth.2020.105585 PMC7553006 33071407

[43] Legal Information Institute. (n.d.). Flowers vs. Jeffords. In Women and justice. Retrieved from https://www.law.cornell.edu/women-and-justice/resource/flowers_v._jeffords

[44] Luthar, S. S., Doernberger, C. H., & Zigler, E. (1993). Resilience is not unidimensional construct: Insights from a prospective study of inner-city adolescents. Development and Psychopathology, 5(4), 703–717. DOI: 10.1017/S0954579400006246 25722542 PMC4339070

[45] Masten, A., & Coatsworth, J. D. (1998). The development of competence in favorable and unfavorable environments: Lessons from research on successful children. American Psychologist, 53(2), 205–220. DOI: 10.1037/0003-066X.53.2.205 9491748

[46] Masten, A. S., Desjardins, C. D., McCormick, C. M., Sally, I., Kuo, C., & Long, J. D. (2010). The significance of childhood competence and problems for adult success in work: A developmental cascade analysis. Development and Psychopathology, 22(3), 679–694. DOI: 10.1017/S0954579410000362 20576187

[47] Masten, A. S., Hubbard, J. J., Gest, S. D., Tellegen, A., Garmezy, N., & Ramirez, M. (1999). Competence in the contest of adversity: Pathways to resilience and maladaptation from childhood to late adolescence. Development and Psychopathology, 11(1), 143–169. DOI: 10.1017/S0954579499001996 10208360

[48] McElrath, K. (2020). Nearly 93% of households with school-age children report some form of distance learning during COVID-19. U.S. Census Bureau. Retrieved from https://www.census.gov/library/stories/2020/08/schooling-during-the-covid-19-pandemic.html

[49] McLoyd, V. C. (1998). Socioeconomic disadvantage and child development. American Psychologist, 53(2), 185–204. DOI: 10.1037/0003-066X.53.2.185 9491747

[50] Palacio, J. O. (2013). Why are Garifuna students underachieving in our primary and secondary schools? Caribbean Quarterly, 59(3/4), 127–146. DOI: 10.1080/00086495.2013.11672501

[51] Pan, X. (2020). Technology acceptance, technological self-efficacy, and attitude toward technology-based self-directed learning: Learning motivation as a mediator. Frontiers in Psychology, 11(564294), 1–11. DOI: 10.3389/fpsyg.2020.564294 33192838 PMC7653185

[52] Patrick, S. W., Henkhaus, L. E., Zickafoose, J. S., Lovell, K., Halvorson, A., Loch, S., Letterie, M., & Davis, M. M. (2020). Well-being of parents and children during the COVID-19 Pandemic: A national survey. Pediatrics, 146(4), 1–8. DOI: 10.1542/peds.2020-016824 32709738

[53] Pollard, J., Hawkins, J. D., & Arthur, M. W. (1999). Risk and protection: Are both necessary to understand diverse behavioral outcomes in adolescence? Social Work Research, 23(3), 145–158. DOI: 10.1093/swr/23.3.145

[54] Pozzoli, T., Gini, G., & Scrimin, S. (2021). Distance learning during the COVID-19 lockdown in Italy: The role of family, school, and individual factors [Advance online publication]. School Psychology, 1–7. DOI: 10.1037/spq0000437 34338538

[55] Rich, G. J., Gentle-Genitty, C., & Estrada, C. (2022). Psychology in Belize. In G. J. Rich & N. A. Ramkumar (Eds.), Psychology in Oceania and the Caribbean (International and Cultural Psychology series, pp. 283–294). Cham: Springer. DOI: 10.1007/978-3-030-87763-7_20

[56] Rosen, M. L., Rodman, A. M., Kasparek, S. W., Mayes, M., Freeman, M. M., Lengua, L. J., Meltzoff, A. N., & McLaughlin, K. A. (2021). Promoting youth mental health during the COVID-19 pandemic: A longitudinal study. PLOS ONE, 16(8), 1–21. DOI: 10.1371/journal.pone.0255294 PMC8357139 34379656

[57] Salkind, N. J. (2010). Encyclopedia of research design (Vols. 1–0). SAGE Publications, Inc. DOI: 10.4135/9781412961288

[58] Sameroff, A. J., & Rosenblum, K. L. (2006). Psychosocial constraints on the development of resilience. Annals of The New York Academy of Sciences, 1094(1), 116–124. DOI: 10.1196/annals.1376.010 17347345

[59] Santibáñez, L., & Guarino, C. M. (2021). The effects of absenteeism on academic and social-emotional outcomes: Lessons for COVID-19. Educational Researcher, 50(6), 392–400. DOI: 10.4135/9781412961288

[60] Scarpellini, F., Segre, G., Cartabia, M., Zanetti, M., Campi, R., Clavenna, A., & Bonati, M. (2021, June 2). Distance learning in Italian primary and middle school children during the COVID-19 pandemic: A national survey. BMC Public Health, 21(1035), 1–13. DOI: 10.1186/s12889-021-11026-x 34078328 PMC8170444

[61] Smokowski, P. R., Mann, E. A., Reynolds, A. J., & Fraser, M. W. (2004). Childhood risk and protective factors and late adolescent adjustment in inner city minority youth. Children and Youth Services Review, 26(1), 63–91. DOI: 10.1016/j.childyouth.2003.11.003

[62] Vairez, M. R., Jr., Hermond, D. S., Gomez, F. C. Jr., & Osho, G. S. (2017). Factors that contribute to the disparity in academic achievement of students from southern Belize. Caribbean Quarterly, 63(1), 83–108. DOI: 10.1080/00086495.2017.1302158

[63] Wilkins, J. (2008). School characteristics that influence student attendance: Experiences of students in a school avoidance program. The High School Journal, 91(3), 12–24. Retrieved from https://muse.jhu.edu/article/232708. DOI: 10.1353/hsj.2008.0005.

[64] Zaccoletti, S., Camacho, A., Correia, N., Aguiar, C., Mason, L., Alves, R. A., & Daniel, J. R. (2020). Parents’ perceptions of student academic motivation during the COVID-19 lockdown: A cross-country comparison. Frontiers in Psychology, 11(592670), 1–13. DOI: 10.3389/fpsyg.2020.592670 33391114 PMC7775314

